# A National Board of Health; Bill in Congress

**Published:** 1891-12

**Authors:** 


### A NATIONAL BOARD OF HEALTH.

Senator Harris, chairman of the Committee on Epidemic Diseases, has introduced a bill in Congress providing for the creation of a National Board of Health, and defining its powers. The JOURNAL acknowledges the courtesy of a copy of the bill. The board is to be under the control of the Treasury Department, and is to consist of seven members. Three members are to be sanitary scientists, and are to be appointed by the President, by and with the consent of the Senate, not more than one to be chosen from any one State. These three members are to receive $5000 salary each. Three medical officers are to be detailed, one from the Army, Navy, and Marine Hospital service respectively, to receive no other compensation than their salary, and the seventh member is to be detailed from the department of justice, and to be learned in the law,—no pay except his salary as judge.

The object of the bill is to better enforce the quarantine laws with reference to the introduction of epidemic diseases from foreign parts.

This is all very well, so far as it goes; but it is a mere sop, even should it pass. What the medical profession want, what the country needs, and what the exigency of the times, common sense and common justice demand, is a Minister of Public Health with a seat in the Cabinet, and powers commensurate with the dignity of the office; to be on a footing, at least, with the Minister of Agriculture. The health of our cattle is carefully looked after by the Agricultural Department, but that of the people—well,—is at the mercy of every quack that has a mind to call himself "doctor."

